# Supplementary material for: Hybrid Models and Biological Model Reduction with PyDSTool
Source: PLoS Comput Biol. 2012 Aug 9;8(8):e1002628. doi: 10.1371/journal.pcbi.1002628 (PMC3415397; doi:10.1371/journal.pcbi.1002628)
Supplement: Text S4 — Complete source code for the PyDSTool package (version 0.88.120504). Includes API documentation and help files linking to web pages. This file is identical to the current public release on Sourceforge.net. (ZIP) [file pcbi.1002628.s004.zip › PyDSTool/html/PyDSTool.Toolbox.phaseplane-module.html]

xml version="1.0" encoding="ascii"?


PyDSTool.Toolbox.phaseplane


| Home | Trees | Indices | Help | PyDSTool |
| --- | --- | --- | --- | --- |

Package PyDSTool ::
Package Toolbox ::
Module phaseplane

[hide private]

[frames] | no frames]

# Module phaseplane

source code

```
    Phase plane utilities.

    Some 2011 functionality has not yet been updated to use the plotter
    phase plane plotting manager.


IMPORTANT NOTE DURING DEVELOPMENT:
For now, many operations with nullclines assume that they are NOT multi-valued
as a function of their variables, and that they are monotonic only as the x
variable increases.

R. Clewley, 2006 - 2011
```


|  |  |
| --- | --- |
| Classes | [hide private] |

distance\_to\_pointset  
First and second maximum and/or minimum distances of a point q
to a set of points, returning a dictionary keyed by 'min' and
'max' to dictionaries keyed by integers 1 and 2 (respectively).

Point2D  
Convenience sub-class of PyDSTool.Point for 2D Euclidean points.

nullcline  
Nullcline representation class in 2D (x, y) plane parameterizable
by x variable only.

fixedpoint\_nD

fixedpoint\_2D

plotter\_2D  
Plotting manager for phase plane analysis.

dx\_scaled\_2D  
Supports a delta x vector that automatically re-scales according to
the known scalings of each of the vector's component directions.

phaseplane  
Working environment for 2D phase-plane analysis.

zone\_node  
Phase plane 'zone' node of hierarchical qualitative feature
abstract class.

zone\_leaf  
Phase plane 'zone' for leaf of hierarchical qualitative feature
abstract class.

nullcline\_zone\_node

nullcline\_zone\_leaf  
Parameters to apply: pars: xtol, refine (integer, default 0)
optional pars: find\_exact\_center (unused)

fixedpoint\_zone

inflection\_zone\_leaf  
A single inflection point zone.

inflection\_zone\_node  
Find all inflection point zones

max\_curvature\_zone\_leaf  
A single zone of locally maximal curvature

max\_curvature\_zone\_node  
Find all zones with locally maximal curvature.

min\_curvature\_zone

mesh\_patch\_2D  
2D mesh patch generator (for 4 or 8 points of a fixed distance from
a central point).

base\_n\_counter  
Simple counter in base-n, using d digits.


|  |  |
| --- | --- |
| Functions | [hide private] |

|  |  |
| --- | --- |
| find\_nullclines(gen, xname, yname, subdomain=None, fps=None, n=10, t=0, eps=None, crop\_tol\_pc=0.01, jac=None, max\_step=0, max\_num\_points=1000, only\_var=None, seed\_points=None, strict\_domains=True, pycont\_cache=None)  Find nullclines of a two-dimensional sub-system of the given Generator object gen, specified by xname and yname. | source code |

|  |  |
| --- | --- |
| find\_fixedpoints(gen, subdomain=None, n=5, maxsearch=1000.0, eps=1e-08, t=0, jac=None)  Find fixed points of a system in a given sub-domain (dictionary), on the assumption that they are isolated points. | source code |

|  |  |
| --- | --- |
| find\_equilibria(gen, subdomain=None, n=5, maxsearch=1000.0, eps=1e-08, t=0, jac=None)  Find fixed points of a system in a given sub-domain (dictionary), on the assumption that they are isolated points. | source code |

|  |  |
| --- | --- |
| find\_steadystates(gen, subdomain=None, n=5, maxsearch=1000.0, eps=1e-08, t=0, jac=None)  Find fixed points of a system in a given sub-domain (dictionary), on the assumption that they are isolated points. | source code |

|  |  |
| --- | --- |
| get\_perp(v)  Find perpendicular vector in 2D (assumes 2D input) | source code |

|  |  |
| --- | --- |
| get\_orthonormal(v)  Returns orthonormal vector in 2D (assumes 2D input) | source code |

|  |  |
| --- | --- |
| get\_rotated(x, theta) | source code |

|  |  |
| --- | --- |
| filter\_close\_points(pts, eps, normord=2)  Remove points from iterable pts (e.g. | source code |

|  |  |
| --- | --- |
| filter\_NaN(pts)  Filter out any points containing NaNs. | source code |

|  |  |
| --- | --- |
| check\_bounds(sol\_array, xinterval, yinterval) | source code |

|  |  |
| --- | --- |
| crop\_2D(sol\_array, xinterval, yinterval)  Filter out points that are outside the domains given by xdom, ydom. | source code |

|  |  |
| --- | --- |
| make\_vec\_at\_A\_face\_B(vec\_at\_a, Ay, By)  Assumes monotonicity of functions on which points A and B lie, using their y components as arguments. | source code |

|  |  |
| --- | --- |
| angle\_to\_vertical(v)  Return an angle between 0 and 2\*pi measured clockwise from vertical. | source code |

|  |  |
| --- | --- |
| is\_counter\_clockwise(A, B, C) | source code |

|  |  |
| --- | --- |
| determine\_if\_intersect(A, B, C, D) | source code |

|  |  |
| --- | --- |
| line\_intersection(A, B, C, D)  Assumes line segments AB vs. | source code |

|  |  |
| --- | --- |
| make\_distance\_to\_known\_line\_auxfn(fname, p, dp=None, q=None)  Builds definition of an auxiliary function of (x,y) measuring (signed) distance from a 2D line given by the point p and either (a) the vector dp, or (b) the point q. | source code |

|  |  |
| --- | --- |
| make\_distance\_to\_line\_auxfn(linename, fname, p, by\_vector\_dp=True)  Builds definition of an auxiliary function of (x,y) measuring (signed) distance from a 2D line given at run-time by coordinate names in the tuple of strings p and either a vector dp, or a point q, depending on the second input argument. | source code |

|  |  |
| --- | --- |
| bisection(func, a, b, args=`(``)`, xtol=1e-10, maxiter=400, normord=2)  Bisection root-finding method. | source code |

|  |  |
| --- | --- |
| \_newton\_step(Q0, nullc, A, B, phi, tol)  Internal function for line intersection involving splines. | source code |

|  |  |
| --- | --- |
| find\_nearest\_sample\_points\_by\_angle(thetas, phi, return\_NaN=False)  Returns two successive indices of the angles in the array argument thetas, for which theta values straddle angle phi. | source code |

|  |  |
| --- | --- |
| closest\_perp\_distance\_between\_sample\_points(NullcA, NullcB, xa, x0B, x1B, newt\_tol=1e-06)  Closest perpendicular distance from (xa, ya) on Nullcline A to Nullcline B, given that it's known to be between sample points x0B and x1B on Nullcline B. | source code |

|  |  |
| --- | --- |
| closest\_perp\_distance\_on\_spline(NullcA, NullcB, xa)  Closest perpendicular distance to Nullcline B at point given by xa on Nullcline A | source code |

|  |  |
| --- | --- |
| is\_min\_bracket(triple)  For a triple of positive numbers (a,b,c), returns a triple (boolean, index, error) | source code |

|  |  |
| --- | --- |
| \_sample\_array\_interior(xdata, pc, refine=0)  xdata is a 1D array. | source code |

|  |  |
| --- | --- |
| closest\_perp\_distance\_between\_splines(NullcA, NullcB, dist\_delta\_tol=1e-05, strict=True)  Measure closest perpendicular distance between two nullcline objects (via their spline representations). | source code |

|  |  |
| --- | --- |
| find\_saddle\_manifolds(fp, dx=None, dx\_gamma=None, dx\_perp=None, tmax=None, max\_len=None, ic=None, ic\_dx=None, max\_pts=1000, directions=`(`1`,` -1`)`, which=`(``'``s``'``,` `'``u``'``)`, other\_pts=None, rel\_scale=None, dx\_perp\_fac=0.75, verboselevel=0, fignum=None)  Compute any branch of the stable or unstable sub-manifolds of a saddle. | source code |

|  |  |
| --- | --- |
| make\_flow\_normal\_event(x, y, dxdt, dydt, targetlang, flatspec=None, fnspec=None, evtArgs=None)  For 2D vector fields only. | source code |

|  |  |
| --- | --- |
| find\_period(pts, thresh, dir=1, with\_indices=False)  pts is a Pointset. | source code |

|  |  |
| --- | --- |
| \_filter\_consecutive(indices, minimize\_values=None)  Return index array containing no consecutive values. | source code |

|  |  |
| --- | --- |
| plot\_PP\_fps(fps, coords=None, do\_evecs=False, markersize=10)  Draw 2D list of fixed points (singletons allowed), must be fixedpoint\_2D objects. | source code |

|  |  |
| --- | --- |
| plot\_PP\_vf(gen, xname, yname, N=20, subdomain=None, scale\_exp=0)  Draw 2D vector field in (xname, yname) coordinates of given Generator, sampling on a uniform grid of n by n points. | source code |

|  |  |
| --- | --- |
| get\_PP(gen, pt, vars, doms=None, doplot=True, t=0, saveplot=None, format=`'``svg``'`, trail\_pts=None, null\_style=`'``-``'`, orbit\_col=`'``g``'`, orbit\_style=`'``-``'`)  Get a phase plane for generator gen at point pt. | source code |

|  |  |
| --- | --- |
| show\_PPs(gen, x, y, traj, t\_start, t\_step, t\_end, moviename=None, doms=None, show\_trail=False, add\_traj=None)  Show phase plane x vs y for Generator gen over trajectory traj, over times t\_start:t\_step:t\_end. | source code |

|  |  |
| --- | --- |
| create\_test\_fn(gen, tmax, dist\_pts)  Create integration test function using the supplied generator. | source code |

|  |  |
| --- | --- |
| create\_test\_fn\_with\_events(gen, tmax, dist\_pts, iso\_ev, other\_evnames, pars\_to\_vars)  Create integration test function using the supplied generator, assuming it contains isochron-related events. | source code |

|  |  |
| --- | --- |
| \_xinf\_ND(xdot, x0, args=`(``)`, xddot=None, xtol=1.49012e-08)  Private function for wrapping the fsolving for x\_infinity for a variable x in N dimensions | source code |

|  |  |
| --- | --- |
| \_xinf\_1D(xdot, x0, args=`(``)`, xddot=None, xtol=1.49012e-08)  Private function for wrapping the solving for x\_infinity for a variable x in 1 dimension | source code |

|  |  |
| --- | --- |
| \_find\_min\_pt(gen, q, pos1, pts, pars\_to\_vars, iso\_ev, other\_evnames, offset=4) | source code |


|  |  |
| --- | --- |
| Variables | [hide private] |

acosh

replaceSepStr

float96

protected\_randomnames

ONES

typeCounter

specialfns

ensuredecimalconst

toCircumflexSyntax

mathlookup

Continuous = `Continuous Domain`

toPowSyntax

rerr = `<PyDSTool.Redirector.Redirector object at 0x4d185b0>`

sym2name

sinh = `<ufunc 'sinh'>`

getlftrtD

doadd

less\_equal = `<ufunc 'less_equal'>`

replaceCallsWithDummies

randomlookup

cPickle

replaceSepQSpec

feval\_map\_const

protected\_auxnamesDB = `ModelSpec internal helper class: auxfnD...`

Inf = `inf`

specTypes = `('RHSfuncSpec', 'ImpFuncSpec', 'ExpFuncSpec')`

logical\_or = `<ufunc 'logical_or'>`

errorfields = `{0: ['t', 'error info'], 10: ['t', 'event list']}`

tan = `<ufunc 'tan'>`

math\_dir

atanh

name\_chars\_RE

temp\_macro\_names\_inv

arccos = `<ufunc 'arccos'>`

isVectorClause

POW\_STR

convert\_power\_reserved\_keywords

W\_NONTERMSTATEBD = `21`

predicate\_op

cosh = `<ufunc 'cosh'>`

inverseMathNameMap = `{'Random': 'Random', 'SystemRandom': 'Sys...`

degrees = `<ufunc 'degrees'>`

greater = `<ufunc 'greater'>`

E\_NONUNIQUETERM = `10`

radians = `<ufunc 'radians'>`

sin = `<ufunc 'sin'>`

symbolMapClass

token

API = `API_class()`

fmod = `<ufunc 'fmod'>`

ensureints

API\_class

funcnames

mathNameMap = `{'Abs': 'abs', 'Acos': 'acos', 'Asin': 'asin', '...`

alphabet\_chars\_RE

re

contained = `contained`

toDoubleStarSyntax

protected\_allnames = `['acos', 'asin', 'atan', 'atan2', 'ceil',...`

ensureparen

math\_globals

ZEROS

reserved\_keywords

arctan2 = `<ufunc 'arctan2'>`

ldexp = `<ufunc 'ldexp'>`

mapNames

trysimple

replaceSep

complex192

temp\_macro\_names

arctanh = `<ufunc 'arctanh'>`

string2ast

W\_UNCERTVAL = `0`

null\_predicate\_class

rout = `<PyDSTool.Redirector.Redirector object at 0x4d18cb0>`

dodiv

parserObject

simplify

isNameToken

W\_TERMSTATEBD = `11`

fit\_function

exp = `<ufunc 'exp'>`

DO\_POW

nameResolver = `ModelSpec internal helper class: nameResolver o...`

DomainType

findEndBrace

frexp = `<ufunc 'frexp'>`

simplify\_str

mapPowStr

ceil = `<ufunc 'ceil'>`

builtin\_auxnames

ensurebare

regObject

targetLangs = `['c', 'python', 'matlab']`

allmathnames = `['acos', 'asin', 'atan', 'atan2', 'ceil', 'cos'...`

isnan = `<ufunc 'isnan'>`

strIfSeq

feval\_map\_symb

isIntegerToken

protected\_specialfns

cos = `<ufunc 'cos'>`

theGenSpecHelper = `GenSpecHelper()`

whoQ

dofun

replaceSepListInv

HAVE\_PSYCO = `True`

replaceSepInv

protected\_mathnames

utils\_info

pi = `3.14159265359`

asinh

arcsin = `<ufunc 'arcsin'>`

doneg

accrueCompTypes

count\_sep

uncertain = `uncertain`

protected\_macronames

tanh = `<ufunc 'tanh'>`

DO\_DEC

builtinlookup

getdim

fabs = `<ufunc 'fabs'>`

NaN = `nan`

sqrt = `<ufunc 'sqrt'>`

complex

ensureparen\_div

LargestInt32 = `2147483647`

allCompTypes

splitargs

splitastLR

dopower

TENS

processMultiDef

W\_NONTERMEVENT = `20`

joinStrs

qtypes

less = `<ufunc 'less'>`

W\_TERMEVENT = `10`

verstr\_parts = `['0', '7', '0']`

errmessages = `{0: 'Computation of trajectory failed', 10: 'Mor...`

W\_BISECTLIMIT = `12`

isNumericToken

isHierarchicalName

genDB = `Generator internal database class: []`

mod = `<ufunc 'remainder'>`

builtinFnSigInfo

warnfields = `{0: ['value', 'interval'], 10: ['t', 'event list'...`

Macheps

hypot = `<ufunc 'hypot'>`

ast2string

domul

remove\_indices\_from\_range

null\_predicate = `null_predicate_class(None)`

collect\_numbers

modf = `<ufunc 'modf'>`

power = `<ufunc 'power'>`

syms

Discrete = `Discrete Domain`

findNumTailPos

makeParList

num\_chars

readArgs

notcontained = `notcontained`

symbolMapDict = `{}`

resolveSpecTypeCombos

arcsinh = `<ufunc 'arcsinh'>`

joinAsStrs

proper\_match

log = `<ufunc 'log'>`

allmathnames\_symbolic = `['Acos', 'Asin', 'Atan', 'Atan2', 'Cei...`

log10 = `<ufunc 'log10'>`

ast2shortlist

modlookup

isToken

replaceSepList

dosub

arccosh = `<ufunc 'arccosh'>`

parser

E\_COMPUTFAIL = `0`

parseMatrixStrToDictStr

warnmessages = `{0: 'Uncertain value computed', 10: 'Terminal e...`

floor = `<ufunc 'floor'>`

local\_fndef

protected\_scipynames

symbol

e = `2.71828182846`

NAMESEP

feature\_leaf

traceback

MReg

dsInterface

process\_raw\_residual

feature\_node

\_seq\_types = `(<type 'list'>, <type 'tuple'>, <type 'numpy.ndar...`

\_num\_types = `(<type 'float'>, <type 'int'>, <type 'numpy.float...`

isfinite = `<ufunc 'isfinite'>`

sign = `<ufunc 'sign'>`

arctan = `<ufunc 'arctan'>`

\_functions = `['find_nullclines', 'make_distance_to_line_auxfn'...`

\_classes = `['distance_to_pointset', 'mesh_patch_2D', 'dx_scale...`

\_features = `['inflection_zone_leaf', 'inflection_zone_node', '...`

plotter = `plotter_2D()`

\_num\_inf = `1e+100`

ALLOW\_THREADS = `1`

Abs = `Abs (ModelSpec wrapper)`

Acos = `Acos (ModelSpec wrapper)`

Asin = `Asin (ModelSpec wrapper)`

Atan = `Atan (ModelSpec wrapper)`

Atan2 = `Atan2 (ModelSpec wrapper)`

BUFSIZE = `10000`

Betavariate = `Betavariate (ModelSpec wrapper)`

CLIP = `0`

Ceil = `Ceil (ModelSpec wrapper)`

Choice = `Choice (ModelSpec wrapper)`

Cos = `Cos (ModelSpec wrapper)`

Cosh = `Cosh (ModelSpec wrapper)`

Degrees = `Degrees (ModelSpec wrapper)`

E = `QuantSpec e (ExpFuncSpec)`

ERR\_CALL = `3`

ERR\_DEFAULT = `0`

ERR\_DEFAULT2 = `2084`

ERR\_IGNORE = `0`

ERR\_LOG = `5`

ERR\_PRINT = `4`

ERR\_RAISE = `2`

ERR\_WARN = `1`

Exp = `Exp (ModelSpec wrapper)`

Expovariate = `Expovariate (ModelSpec wrapper)`

FLOATING\_POINT\_SUPPORT = `1`

FPE\_DIVIDEBYZERO = `1`

FPE\_INVALID = `8`

FPE\_OVERFLOW = `2`

FPE\_UNDERFLOW = `4`

Fabs = `Fabs (ModelSpec wrapper)`

False\_ = `False`

Floor = `Floor (ModelSpec wrapper)`

Fmod = `Fmod (ModelSpec wrapper)`

Frexp = `Frexp (ModelSpec wrapper)`

Gammavariate = `Gammavariate (ModelSpec wrapper)`

Gauss = `Gauss (ModelSpec wrapper)`

Getrandbits = `Getrandbits (ModelSpec wrapper)`

Getstate = `Getstate (ModelSpec wrapper)`

Hypot = `Hypot (ModelSpec wrapper)`

Infinity = `inf`

Jumpahead = `Jumpahead (ModelSpec wrapper)`

Ldexp = `Ldexp (ModelSpec wrapper)`

Log = `Log (ModelSpec wrapper)`

Log10 = `Log10 (ModelSpec wrapper)`

Lognormvariate = `Lognormvariate (ModelSpec wrapper)`

MAXDIMS = `32`

Max = `Max (ModelSpec wrapper)`

Min = `Min (ModelSpec wrapper)`

Modf = `Modf (ModelSpec wrapper)`

NAN = `nan`

NINF = `-inf`

NZERO = `-0.0`

Normalvariate = `Normalvariate (ModelSpec wrapper)`

PINF = `inf`

PZERO = `0.0`

Paretovariate = `Paretovariate (ModelSpec wrapper)`

Pi = `QuantSpec pi (ExpFuncSpec)`

Pow = `Pow (ModelSpec wrapper)`

RAISE = `2`

Radians = `Radians (ModelSpec wrapper)`

Randint = `Randint (ModelSpec wrapper)`

Random = `Random (ModelSpec wrapper)`

Randrange = `Randrange (ModelSpec wrapper)`

SHIFT\_DIVIDEBYZERO = `0`

SHIFT\_INVALID = `9`

SHIFT\_OVERFLOW = `3`

SHIFT\_UNDERFLOW = `6`

Sample = `Sample (ModelSpec wrapper)`

ScalarType = `(<type 'int'>, <type 'float'>, <type 'complex'>, ...`

Seed = `Seed (ModelSpec wrapper)`

Setstate = `Setstate (ModelSpec wrapper)`

Shuffle = `Shuffle (ModelSpec wrapper)`

Sin = `Sin (ModelSpec wrapper)`

Sinh = `Sinh (ModelSpec wrapper)`

Sqrt = `Sqrt (ModelSpec wrapper)`

Sum = `Sum (ModelSpec wrapper)`

Systemrandom = `Systemrandom (ModelSpec wrapper)`

Tan = `Tan (ModelSpec wrapper)`

Tanh = `Tanh (ModelSpec wrapper)`

True\_ = `True`

UFUNC\_BUFSIZE\_DEFAULT = `10000`

UFUNC\_PYVALS\_NAME = `'UFUNC_PYVALS'`

Uniform = `Uniform (ModelSpec wrapper)`

Vonmisesvariate = `Vonmisesvariate (ModelSpec wrapper)`

WRAP = `1`

Weibullvariate = `Weibullvariate (ModelSpec wrapper)`

Wichmannhill = `Wichmannhill (ModelSpec wrapper)`

absolute = `<ufunc 'absolute'>`

add = `<ufunc 'add'>`

bitwise\_and = `<ufunc 'bitwise_and'>`

bitwise\_not = `<ufunc 'invert'>`

bitwise\_or = `<ufunc 'bitwise_or'>`

bitwise\_xor = `<ufunc 'bitwise_xor'>`

c\_ = `<numpy.lib.index_tricks.CClass object at 0x1154330>`

cast = `{<type 'numpy.int64'>: <function <lambda> at 0x10a8bb0>...`

conj = `<ufunc 'conjugate'>`

conjugate = `<ufunc 'conjugate'>`

copysign = `<ufunc 'copysign'>`

deg2rad = `<ufunc 'deg2rad'>`

divide = `<ufunc 'divide'>`

equal = `<ufunc 'equal'>`

exp2 = `<ufunc 'exp2'>`

expm1 = `<ufunc 'expm1'>`

floor\_divide = `<ufunc 'floor_divide'>`

fmax = `<ufunc 'fmax'>`

fmin = `<ufunc 'fmin'>`

greater\_equal = `<ufunc 'greater_equal'>`

index\_exp = `<numpy.lib.index_tricks.IndexExpression object at ...`

inf = `inf`

infty = `inf`

invert = `<ufunc 'invert'>`

isinf = `<ufunc 'isinf'>`

left\_shift = `<ufunc 'left_shift'>`

little\_endian = `True`

log1p = `<ufunc 'log1p'>`

logaddexp = `<ufunc 'logaddexp'>`

logaddexp2 = `<ufunc 'logaddexp2'>`

logical\_and = `<ufunc 'logical_and'>`

logical\_not = `<ufunc 'logical_not'>`

logical\_xor = `<ufunc 'logical_xor'>`

maximum = `<ufunc 'maximum'>`

mgrid = `<numpy.lib.index_tricks.nd_grid object at 0x113d490>`

minimum = `<ufunc 'minimum'>`

multiply = `<ufunc 'multiply'>`

n = `9`

nan = `nan`

nbytes = `{<type 'numpy.int64'>: 8, <type 'numpy.int16'>: 2, <t...`

negative = `<ufunc 'negative'>`

newaxis = `None`

nextafter = `<ufunc 'nextafter'>`

not\_equal = `<ufunc 'not_equal'>`

ogrid = `<numpy.lib.index_tricks.nd_grid object at 0x113dbb0>`

ones\_like = `<ufunc 'ones_like'>`

r\_ = `<numpy.lib.index_tricks.RClass object at 0x11542f0>`

rad2deg = `<ufunc 'rad2deg'>`

reciprocal = `<ufunc 'reciprocal'>`

remainder = `<ufunc 'remainder'>`

right\_shift = `<ufunc 'right_shift'>`

rint = `<ufunc 'rint'>`

s\_ = `<numpy.lib.index_tricks.IndexExpression object at 0x11543f0>`

sctypeDict = `{0: <type 'numpy.bool_'>, 1: <type 'numpy.int8'>,...`

sctypeNA = `{'?': 'Bool', 'B': 'UInt8', 'Bool': <type 'numpy.bo...`

sctypes = `{'complex': [<type 'numpy.complex64'>, <type 'numpy....`

signbit = `<ufunc 'signbit'>`

spacing = `<ufunc 'spacing'>`

square = `<ufunc 'square'>`

subtract = `<ufunc 'subtract'>`

t = `'0'`

true\_divide = `<ufunc 'true_divide'>`

trunc = `<ufunc 'trunc'>`

typeDict = `{0: <type 'numpy.bool_'>, 1: <type 'numpy.int8'>, 2...`

typeNA = `{'?': 'Bool', 'B': 'UInt8', 'Bool': <type 'numpy.bool...`

typecodes = `{'All': '?bhilqpBHILQPfdgFDGSUVOMm', 'AllFloat': '...`


|  |  |
| --- | --- |
| Function Details | [hide private] |

|  |  |
| --- | --- |
| find\_nullclines(gen, xname, yname, subdomain=None, fps=None, n=10, t=0, eps=None, crop\_tol\_pc=0.01, jac=None, max\_step=0, max\_num\_points=1000, only\_var=None, seed\_points=None, strict\_domains=True, pycont\_cache=None) | source code |

```
Find nullclines of a two-dimensional sub-system of the given
Generator object gen, specified by xname and yname.

Inputs:

gen is a Generator object
xname, yname are state variable names of gen

Optional inputs:

Restriction of x and y to sub-domains can be made using the subdomain argument.
subdomain may contain pairs (min, max) or singleton values that fix those state
variables and restrict the fixed point search to the remaining sub-system on the
given ranges. (default to domains given in generator). There must be exactly
two ranges remaining (for xname, yname) to give a two-dimensional nullcline
problem, unless only_var option has been used (see below).

n = initial number of meshpoints for fsolve. Do not set this large if using
  PyCont, e.g. use n=3. Default is 10.

Set t value for non-autonomous systems (default 0). Support for Jacobians
  with non-autonomous systems is not yet provided.

jac is a Jacobian function that accepts keyword arguments including t for
  time (even if Jacobian is time-independent).

max_step (dictionary) tells PyCont the largest step size to use for each
  variable. Integer 0 (default) switches off PyCont use. Use None
  to tell PyCont to use default max step size (5e-1).

fps can be a list of points previously calculated to be fixed points,
  which this function will use as additional starting points for
  computation.

eps sets the accuracy to which the nullclines are calculated. Default is
  approximately 1e-8.

crop_tol_pc is the percentage (default 1%) for tolerance outside the specified
  domain for keeping points in nullclines -- final points will be cropped with
  this tolerance.

only_var (variable name) requests that only the nullcline for that variable
  will be computed. The variable name must correspond to xname or yname.

seed_points can be used if an approximate position on either nullcline is already
  known, especially useful if there are no fixed points provided. One or more
  seed points should be given as a list of dictionaries or Points with keys or
  coordinates xname and yname. These should be collected as values of the
  seed_points dictionary, whose keys (xname and/or yname) indicate which points
  belong to which nullclines.

strict_domains (boolean, default True) ensures all computed nullcline points are
  cropped to lie within the given sub-domains.

pycont_cache (list of two PyCont objects, default None) can be provided to
  improve efficiency of PyCont use, to avoid re-creation of ODE objects for
  each call to this function. If a list of [None, None] is provided, this will
  ensure that a list of the two PyCont objects will be returned in an additional
  output. !!Don't use this cache if external inputs to the Generator have changed!!

Output:
    (x_null, y_null) arrays of (x,y) pairs. Fixed points will be included if
    they are provided (they are expected to be calculated to high enough
    accuracy to smoothly fit with the nullcline sample points).

    (optional) [P_x, P_y] list of PyCont objects, returned if
      pycont_cache input option is used (see above).

Note that the points returned are not guaranteed to be in any particular
order when PyCont is not used.
```

|  |  |
| --- | --- |
| find\_fixedpoints(gen, subdomain=None, n=5, maxsearch=1000.0, eps=1e-08, t=0, jac=None) | source code |

Find fixed points of a system in a given sub-domain (dictionary), on
the assumption that they are isolated points. subdomain may contain pairs
(min, max) or singleton values that fix those state variables and
restrict the fixed point search to the remaining sub-system on the given
ranges. (default to domains given in generator).

Returns list of dictionaries mapping the variable names to the
values.

Set t value for non-autonomous systems (default 0).

|  |  |
| --- | --- |
| find\_equilibria(gen, subdomain=None, n=5, maxsearch=1000.0, eps=1e-08, t=0, jac=None) | source code |

Find fixed points of a system in a given sub-domain (dictionary), on
the assumption that they are isolated points. subdomain may contain pairs
(min, max) or singleton values that fix those state variables and
restrict the fixed point search to the remaining sub-system on the given
ranges. (default to domains given in generator).

Returns list of dictionaries mapping the variable names to the
values.

Set t value for non-autonomous systems (default 0).

|  |  |
| --- | --- |
| find\_steadystates(gen, subdomain=None, n=5, maxsearch=1000.0, eps=1e-08, t=0, jac=None) | source code |

Find fixed points of a system in a given sub-domain (dictionary), on
the assumption that they are isolated points. subdomain may contain pairs
(min, max) or singleton values that fix those state variables and
restrict the fixed point search to the remaining sub-system on the given
ranges. (default to domains given in generator).

Returns list of dictionaries mapping the variable names to the
values.

Set t value for non-autonomous systems (default 0).

|  |  |
| --- | --- |
| filter\_close\_points(pts, eps, normord=2) | source code |

Remove points from iterable pts (e.g. array or Pointset) according to
whether they are closer than epsilon to each other in the given norm.

|  |  |
| --- | --- |
| filter\_NaN(pts) | source code |

Filter out any points containing NaNs. Expects array input.

|  |  |
| --- | --- |
| crop\_2D(sol\_array, xinterval, yinterval) | source code |

Filter out points that are outside the domains given by xdom, ydom.
Expects array input

|  |  |
| --- | --- |
| line\_intersection(A, B, C, D) | source code |

Assumes line segments AB vs. CD actually cross (must determine this
separately), where points A - D have fields 'x' and 'y' e.g. a Point2D
object.

Uses algorithm from:
http://www.bryceboe.com/2006/10/23/line-segment-intersection-algorithm/
and http://paulbourke.net/geometry/lineline2d/

In particular, this code assumes lines are not parallel or
collinear.

|  |  |
| --- | --- |
| make\_distance\_to\_known\_line\_auxfn(fname, p, dp=None, q=None) | source code |

Builds definition of an auxiliary function of (x,y) measuring (signed)
distance from a 2D line given by the point p and either (a) the vector
dp, or (b) the point q. (All inputs must be Points.)

|  |  |
| --- | --- |
| make\_distance\_to\_line\_auxfn(linename, fname, p, by\_vector\_dp=True) | source code |

Builds definition of an auxiliary function of (x,y) measuring (signed)
distance from a 2D line given at run-time by coordinate names in the
tuple of strings p and either a vector dp, or a point q, depending on the
second input argument. Also returns list of parameter names used.

|  |  |
| --- | --- |
| bisection(func, a, b, args=`(``)`, xtol=1e-10, maxiter=400, normord=2) | source code |

Bisection root-finding method. Given a function returning +/- 1 and
an interval with func(a) \* func(b) < 0, find the root between a and
b.

Variant of scipy.optimize.minpack.bisection with exception for too
many iterations

|  |  |
| --- | --- |
| \_newton\_step(Q0, nullc, A, B, phi, tol) | source code |

Internal function for line intersection involving splines. Q0 is the
initial point on the given nullcline. A closer approximation Q on the
nullcline where it intersects line AB

|  |  |
| --- | --- |
| find\_nearest\_sample\_points\_by\_angle(thetas, phi, return\_NaN=False) | source code |

```
Returns two successive indices of the angles in the array argument
thetas, for which theta values straddle angle phi.

This function is useful if phi represents the angle from the vertical of
the normal to a curve's tangent line, and thetas are angles from the
vertical of another curve in the plane. This function can therefore help
find closest or furthest perpendicular distances between curves.

Assumes thetas are in increasing order, and that phi is properly contained
  between min(thetas) and max(thetas) (otherwise raises a ValueError).

For the corner case where one difference between angles is exactly 0 (not
  generic), one of the thetas values given by the returned indices is
  exactly phi.
```

|  |  |
| --- | --- |
| closest\_perp\_distance\_between\_sample\_points(NullcA, NullcB, xa, x0B, x1B, newt\_tol=1e-06) | source code |

Closest perpendicular distance from (xa, ya) on Nullcline A to
Nullcline B, given that it's known to be between sample points x0B and
x1B on Nullcline B.

Uses a Newton-like step, solving up to an angular tolerance given by
newt\_tol.

|  |  |
| --- | --- |
| is\_min\_bracket(triple) | source code |

For a triple of positive numbers (a,b,c), returns a triple (boolean,
index, error)

where: boolean is True if a > b < c (this brackets a minimum
value);

index is 1 (middle) if input brackets a minimum value, otherwise index
indicates which end of the bracket has the smallest value;

error is the smallest absolute difference between distances in the
triple (a, b, c)

|  |  |
| --- | --- |
| closest\_perp\_distance\_between\_splines(NullcA, NullcB, dist\_delta\_tol=1e-05, strict=True) | source code |

Measure closest perpendicular distance between two nullcline objects
(via their spline representations).

Tolerance argument measures that of Euclidean distance between the
splines representing the two nullclines.

strict option (default True) ensures both nullclines are monotonically
increasing or decreasing functions of x. If False, on nullcline A must
be, while nullcline B non-monotonicity will simply lead to a warning
message (since B plays an asymmetric role to A in the calculation, its
monotonicity is less critical).

Assumes monotonicity of both nullclines, which may be relaxed in
future versions.

|  |  |
| --- | --- |
| find\_saddle\_manifolds(fp, dx=None, dx\_gamma=None, dx\_perp=None, tmax=None, max\_len=None, ic=None, ic\_dx=None, max\_pts=1000, directions=`(`1`,` -1`)`, which=`(``'``s``'``,` `'``u``'``)`, other\_pts=None, rel\_scale=None, dx\_perp\_fac=0.75, verboselevel=0, fignum=None) | source code |

```
Compute any branch of the stable or unstable sub-manifolds of a saddle.
Accepts fixed point instances of class fixedpoint_2D.

Required inputs:
  fp:       fixed point object
  dx:       arc-length step size (**fixed**)
  dx_gamma: determines the positions of the Gamma_plus and Gamma_minus
            event surfaces (can be a real scalar or a pair if not symmetric)
  dx_perp:  initial perturbation from the local linear sub-manifolds to
            find starting points.
  tmax:     maximum time to compute a trajectory before 'failing'
  max_len:  maximum arc length to compute
  max_pts:  maximum number of points to compute on each sub-manifold branch
  Specify either ic or ic_dx for initial point (e.g. to restart the calc
    after a previous failure) or a certain distance from the saddle point.

Optional inputs:
  rel_scale:  a pair giving relative scalings of x and y coordinates in
    the plane, to improve stepping in the different directions.
    e.g. (1,10) would make dx steps in the y-direction 10 times larger than
    in the x-direction.

  which:  which sub-manifold to compute 's', 'u' or ('s', 'u').
    Default is both.

  directions:  which directions along chosen sub-manifolds? (1,), (-1,)
    or (1,-1). Default is both.

  other_pts can be a list of points whose proximity will be checked,
and the computation halted if they get within dx of the manifold.

  dx_perp_fac:  For advanced use only. If you get failures saying dx_perp
     too small and that initial displacement did not straddle manifold, try
     increasing this factor towards 1 (default 0.75). Especially for
     unstable manifolds, initial values for dx_perp may diverge, but if
     dx_perp is shrunk too quickly with this factor the sweet spot may be
     missed.

  verboselevel
  fignum
```

|  |  |
| --- | --- |
| make\_flow\_normal\_event(x, y, dxdt, dydt, targetlang, flatspec=None, fnspec=None, evtArgs=None) | source code |

For 2D vector fields only.

Supply flatspec if built vector field using ModelConstructor tools,
otherwise specify funcspec argument.

|  |  |
| --- | --- |
| find\_period(pts, thresh, dir=1, with\_indices=False) | source code |

pts is a Pointset.

thresh is a 1D Point or a dictionary, whose coordinate name
corresponds to a variable in the pts pointset.

dir is either 1 or -1, indicating which direction the threshold must
be crossed to be counted (default 1 = increasing).

with\_indices is a Boolean indicating whether to return the pair of
indices indicating the beginning and end points of the last period
(default False).

|  |  |
| --- | --- |
| \_filter\_consecutive(indices, minimize\_values=None) | source code |

```
Return index array containing no consecutive values.
if minimize_values option is given, this sequence must contain positions for
all indices in the first argument, and will be used to choose which of any
consecutive indices to retain in the returned sequence. Otherwise, the first
index will be chosen arbitrarily.

E.g. to find the *last* index of every cluster instead of the default, use
  minimize_values=range(max_index,0,-1)
```

|  |  |
| --- | --- |
| plot\_PP\_fps(fps, coords=None, do\_evecs=False, markersize=10) | source code |

Draw 2D list of fixed points (singletons allowed), must be
fixedpoint\_2D objects.

Optional do\_evecs (default False) draws eigenvectors around each
f.p.

Requires matplotlib

|  |  |
| --- | --- |
| plot\_PP\_vf(gen, xname, yname, N=20, subdomain=None, scale\_exp=0) | source code |

```
Draw 2D vector field in (xname, yname) coordinates of given Generator,
sampling on a uniform grid of n by n points.

Optional subdomain dictionary specifies axes limits in each variable,
otherwise Generator's xdomain attribute will be used.

For systems of dimension > 2, the non-phase plane variables will be held
  constant at their initial condition values set in the Generator.

Optional scale_exp is an exponent (domain is all reals) which rescales
  size of arrows in case of disparate scales in the vector field. Larger
  values of scale magnify the arrow sizes. For stiff vector fields, values
  from -3 to 3 may be necessary to resolve arrows in certain regions.

Requires matplotlib 0.99 or later
```

|  |  |
| --- | --- |
| get\_PP(gen, pt, vars, doms=None, doplot=True, t=0, saveplot=None, format=`'``svg``'`, trail\_pts=None, null\_style=`'``-``'`, orbit\_col=`'``g``'`, orbit\_style=`'``-``'`) | source code |

Get a phase plane for generator gen at point pt. Specify t if the
system is non-autonomous. If trail\_pts are given (e.g., via show\_PPs)
then these are added behind the point pt.

Requires matplotlib

|  |  |
| --- | --- |
| show\_PPs(gen, x, y, traj, t\_start, t\_step, t\_end, moviename=None, doms=None, show\_trail=False, add\_traj=None) | source code |

Show phase plane x vs y for Generator gen over trajectory traj, over
times t\_start:t\_step:t\_end. Option to create a movie, provided ffmpeg if
installed. Optional domain dictionary for variables can be provided,
otherwise their extents in the given trajectory will be used. Option to
show trail behind green orbit (default False). Option to add a comparison
trajectory / nullclines to the plot.

Requires matplotlib and ffmpeg

|  |  |
| --- | --- |
| create\_test\_fn(gen, tmax, dist\_pts) | source code |

Create integration test function using the supplied generator.

The test function will return a dictionary of the two closest points
(keys 1 and 2) mapping to their respective distances and pointset index
positions.

|  |  |
| --- | --- |
| create\_test\_fn\_with\_events(gen, tmax, dist\_pts, iso\_ev, other\_evnames, pars\_to\_vars) | source code |

Create integration test function using the supplied generator,
assuming it contains isochron-related events.

The test function will return a dictionary of the two closest points
(keys 1 and 2) mapping to their respective distances and pointset index
positions.

  


|  |  |
| --- | --- |
| Variables Details | [hide private] |

### protected\_auxnamesDB

Value:
:   ```
    ModelSpec internal helper class: auxfnDBclass object
    ```

### inverseMathNameMap

Value:
:   ```
    {'Random': 'Random',
     'SystemRandom': 'Systemrandom',
     'WichmannHill': 'Wichmannhill',
     'abs': 'Abs',
     'acos': 'Acos',
     'asin': 'Asin',
     'atan': 'Atan',
     'atan2': 'Atan2',
    ...
    ```

### mathNameMap

Value:
:   ```
    {'Abs': 'abs',
     'Acos': 'acos',
     'Asin': 'asin',
     'Atan': 'atan',
     'Atan2': 'atan2',
     'Betavariate': 'betavariate',
     'Ceil': 'ceil',
     'Choice': 'choice',
    ...
    ```

### protected\_allnames

Value:
:   ```
    ['acos',
     'asin',
     'atan',
     'atan2',
     'ceil',
     'cos',
     'cosh',
     'degrees',
    ...
    ```

### nameResolver

Value:
:   ```
    ModelSpec internal helper class: nameResolver object
    ```

### allmathnames

Value:
:   ```
    ['acos',
     'asin',
     'atan',
     'atan2',
     'ceil',
     'cos',
     'cosh',
     'degrees',
    ...
    ```

### errmessages

Value:
:   ```
    {0: 'Computation of trajectory failed',
     10: 'More than one terminal event found'}
    ```

### warnfields

Value:
:   ```
    {0: ['value', 'interval'],
     10: ['t', 'event list'],
     11: ['t', 'var name', 'var value', '\n\tvalue interval'],
     12: ['t', 'event list'],
     20: ['t', 'event list'],
     21: ['t', 'var name', 'var value', '\n\tvalue interval']}
    ```

### allmathnames\_symbolic

Value:
:   ```
    ['Acos',
     'Asin',
     'Atan',
     'Atan2',
     'Ceil',
     'Cos',
     'Cosh',
     'Degrees',
    ...
    ```

### warnmessages

Value:
:   ```
    {0: 'Uncertain value computed',
     10: 'Terminal event(s) found',
     11: 'State variable reached bounds (terminal)',
     12: 'Bisection limit reached for event',
     20: 'Non-terminal event(s) found',
     21: 'State or input variable reached bounds (non-terminal)'}
    ```

### \_seq\_types

Value:
:   ```
    (<type 'list'>, <type 'tuple'>, <type 'numpy.ndarray'>)
    ```

### \_num\_types

Value:
:   ```
    (<type 'float'>,
     <type 'int'>,
     <type 'numpy.floating'>,
     <type 'numpy.integer'>)
    ```

### \_functions

Value:
:   ```
    ['find_nullclines',
     'make_distance_to_line_auxfn',
     'make_distance_to_known_line_auxfn',
     'crop_2D',
     'find_period',
     'make_flow_normal_event',
     'filter_NaN',
     'find_fixedpoints',
    ...
    ```

### \_classes

Value:
:   ```
    ['distance_to_pointset',
     'mesh_patch_2D',
     'dx_scaled_2D',
     'phaseplane',
     'fixedpoint_nD',
     'fixedpoint_2D',
     'nullcline',
     'Point2D',
    ...
    ```

### \_features

Value:
:   ```
    ['inflection_zone_leaf',
     'inflection_zone_node',
     'max_curvature_zone_leaf',
     'max_curvature_zone_node']
    ```

### ScalarType

Value:
:   ```
    (<type 'int'>,
     <type 'float'>,
     <type 'complex'>,
     <type 'long'>,
     <type 'bool'>,
     <type 'str'>,
     <type 'unicode'>,
     <type 'buffer'>,
    ...
    ```

### cast

Value:
:   ```
    {<type 'numpy.int64'>: <function <lambda> at 0x10a8bb0>, <type 'numpy.
    int16'>: <function <lambda> at 0x10a8bf0>, <type 'numpy.complex128'>: 
    <function <lambda> at 0x10a8c30>, <type 'numpy.uint64'>: <function <la
    mbda> at 0x10a8c70>, <type 'numpy.complex256'>: <function <lambda> at 
    0x10a8cf0>, <type 'numpy.float32'>: <function <lambda> at 0x10a8d30>, 
    <type 'numpy.bool_'>: <function <lambda> at 0x10a8cb0>, <type 'numpy.u
    int8'>: <function <lambda> at 0x10a8db0>, <type 'numpy.int32'>: <funct
    ion <lambda> at 0x10a8eb0>, <type 'numpy.int8'>: <function <lambda> at
    ...
    ```

### index\_exp

Value:
:   ```
    <numpy.lib.index_tricks.IndexExpression object at 0x11543b0>
    ```

### nbytes

Value:
:   ```
    {<type 'numpy.int64'>: 8, <type 'numpy.int16'>: 2, <type 'numpy.comple
    x128'>: 16, <type 'numpy.uint64'>: 8, <type 'numpy.bool_'>: 1, <type '
    numpy.complex256'>: 32, <type 'numpy.float32'>: 4, <type 'numpy.int8'>
    : 1, <type 'numpy.uint8'>: 1, <type 'numpy.uint16'>: 2, <type 'numpy.o
    bject_'>: 4, <type 'numpy.float64'>: 8, <type 'numpy.int32'>: 4, <type
     'numpy.string_'>: 0, <type 'numpy.void'>: 0, <type 'numpy.float128'>:
     16, <type 'numpy.int32'>: 4, <type 'numpy.uint32'>: 4, <type 'numpy.u
    nicode_'>: 0, <type 'numpy.complex64'>: 8, <type 'numpy.uint32'>: 4}
    ```

### sctypeDict

Value:
:   ```
    {0: <type 'numpy.bool_'>,
     1: <type 'numpy.int8'>,
     2: <type 'numpy.uint8'>,
     3: <type 'numpy.int16'>,
     4: <type 'numpy.uint16'>,
     5: <type 'numpy.int32'>,
     6: <type 'numpy.uint32'>,
     7: <type 'numpy.int32'>,
    ...
    ```

### sctypeNA

Value:
:   ```
    {'?': 'Bool',
     'B': 'UInt8',
     'Bool': <type 'numpy.bool_'>,
     'Complex128': <type 'numpy.complex256'>,
     'Complex32': <type 'numpy.complex64'>,
     'Complex64': <type 'numpy.complex128'>,
     'D': 'Complex64',
     'F': 'Complex32',
    ...
    ```

### sctypes

Value:
:   ```
    {'complex': [<type 'numpy.complex64'>,
                 <type 'numpy.complex128'>,
                 <type 'numpy.complex256'>],
     'float': [<type 'numpy.float32'>,
               <type 'numpy.float64'>,
               <type 'numpy.float128'>],
     'int': [<type 'numpy.int8'>,
             <type 'numpy.int16'>,
    ...
    ```

### typeDict

Value:
:   ```
    {0: <type 'numpy.bool_'>,
     1: <type 'numpy.int8'>,
     2: <type 'numpy.uint8'>,
     3: <type 'numpy.int16'>,
     4: <type 'numpy.uint16'>,
     5: <type 'numpy.int32'>,
     6: <type 'numpy.uint32'>,
     7: <type 'numpy.int32'>,
    ...
    ```

### typeNA

Value:
:   ```
    {'?': 'Bool',
     'B': 'UInt8',
     'Bool': <type 'numpy.bool_'>,
     'Complex128': <type 'numpy.complex256'>,
     'Complex32': <type 'numpy.complex64'>,
     'Complex64': <type 'numpy.complex128'>,
     'D': 'Complex64',
     'F': 'Complex32',
    ...
    ```

### typecodes

Value:
:   ```
    {'All': '?bhilqpBHILQPfdgFDGSUVOMm',
     'AllFloat': 'fdgFDG',
     'AllInteger': 'bBhHiIlLqQpP',
     'Character': 'c',
     'Complex': 'FDG',
     'Datetime': 'Mm',
     'Float': 'fdg',
     'Integer': 'bhilqp',
    ...
    ```

  


| Home | Trees | Indices | Help | PyDSTool |
| --- | --- | --- | --- | --- |

|  |  |
| --- | --- |
| Generated by Epydoc 3.0.1 on Fri May 4 15:24:05 2012 | http://epydoc.sourceforge.net |
